# A Rapid Colorimetric Assay for On-Site Authentication of Cephalopod Species

**DOI:** 10.3390/bios10120190

**Published:** 2020-11-24

**Authors:** Giuseppina Tatulli, Paola Cecere, Davide Maggioni, Andrea Galimberti, Pier Paolo Pompa

**Affiliations:** 1 Istituto Italiano di Tecnologia, Nanobiointeractions&Nanodiagnostics, Via Morego 30, 16163 Genova, Italy; giuseppina.tatulli@iit.it (G.T.); paola.cecere@iit.it (P.C.); 2 Department of Earth and Environmental Sciences (DISAT), University of Milano-Bicocca, P.za Della Scienza 1, 20126 Milan, Italy; davide.maggioni@unimib.it; 3 Marine Research and High Education (MaRHE) Center, University of Milano-Bicocca, Faafu Magoodhoo 12030, Maldives; 4 ZooPlantLab, Department of Biotechnology and Biosciences, University of Milano-Bicocca, P.za Della Scienza 2, 20126 Milan, Italy

**Keywords:** colorimetric test, on-site test, LAMP, food authentication, genetic traceability, cephalopod species

## Abstract

A colorimetric assay, exploiting the combination of loop-mediated isothermal amplification (LAMP) with DNA barcoding, was developed to address the authentication of some cephalopod species, a relevant group in the context of seafood traceability, due to the intensive processing from the fishing sites to the shelf. The discriminating strategy relies on accurate design of species-specific LAMP primers within the conventional 5’ end of the mitochondrial COI DNA barcode region and allows for the identification of *Loligo vulgaris* among two closely related and less valuable species. The assay, coupled to rapid genomic DNA extraction, is suitable for large-scale screenings and on-site applications due to its easy procedures, with fast (30 min) and visual readout.

## 1. Introduction

Food adulteration has become a concern of great interest worldwide. This issue is strictly connected to the phenomenon of trade globalization and overexploitation of biodiversity, which are causing profound changes in the whole food supply chain and market systems. Seafood is among the most adulterated food categories [1]. Its long and complex production chain from catching to filleting includes several steps, making fish, crustacean and mollusk products highly exposed to the risk of fraud events [2]. To ensure transparent information to the consumers, food authentication and traceability along the food chain have become essential issues to be addressed. In this framework, the development of rapid, low-cost, and easy-to-use biosensing technologies for on-site diagnostics would be of great and urgent importance. Currently, DNA-based techniques are the most widely used and have proven very effective, especially in combination with the DNA barcoding approach [3,4,5]. DNA barcoding is a method of species identification that is based on the variability analysis of short genomic sequences called DNA barcodes (e.g., the 658 bp 5’ end portion of the mitochondrial gene cytochrome c oxidase subunit I - COI, used for metazoans identification [6,7,8]) that are reported in a unique database called the Barcode of Life Data (BOLD) System [9]. PCR-based methods targeting a DNA barcode sequence have provided several advantages, due to their high discriminating power and sensitivity [10,11,12]. Nevertheless, their requirements for long procedures and complex instrumentation make them unsuitable for rapid and portable testing. In an attempt to simplify the overall procedures, nanotechnologies have offered new perspectives in developing DNA barcoding-based hybrid techniques with naked-eye colorimetric readout, taking advantage of the plasmonic properties of gold nanoparticles [13,14]. A possible advancement toward the development of faster and portable technologies for on-site testing is represented by isothermal amplification techniques. Generally, such reactions work in isothermal conditions (usually 55–65 °C), are very rapid (less than 1 h), and yield amplification efficiencies comparable to the standard PCR [15]. Loop-mediated isothermal amplification (LAMP) was first introduced by Notomi [16] and, due to its rapid and specific DNA amplification in isothermal conditions, is currently one of the most applied techniques in several fields [17,18,19,20,21], including food authentication [22,23,24,25]. This robust methodology uses a DNA polymerase enzyme with strand displacement activity and four to six primers that recognize six to eight regions on the nucleic acid target [16,26]. Recently, the classical analysis of LAMP products by gel electrophoresis has been substituted by real-time [27,28] and naked-eye readout [29,30,31], including the colorimetric method based on pH-sensitive dyes [20,32]. Therefore, LAMP reaction, if performed with portable simple instrumentation such as a heating block, is suitable to realize rapid, one-step, and on-site applications. Here, we present the development of a rapid colorimetric assay combining LAMP with DNA barcoding to address naked-eye authentication of food species. In particular, we applied this approach to a real scenario of seafood fraud, i.e., the authentication of the cephalopod mollusk *Loligo vulgaris* and its discrimination from two genetically similar and often mislabeled species, *Loligo reynaudii* and *Loligo forbesii* [14]. This one-step assay enables the visualization of the colorimetric result within 30 min, also when applied to crude extracts by rapid DNA extraction protocols.

## 2. Materials and Methods

### 2.1. Sampling, Dataset Assembling for Primers Design, and DNA Extraction

*Loligo* spp. tissues were obtained from a previous study assessing the mislabeling rate of cephalopod seafood items in the Italian market using a DNA barcoding approach [14]. COI sequences of the analyzed samples, together with other sequences of *Loligo vulgaris*, *L. forbesii*, *L. reynaudii*, and closely related species (total number of sequences = 140), were downloaded from GenBank and aligned using MUSCLE online [33] to perform LAMP primer design. Similarly, 16S ribosomal RNA sequences of the same species (total number of sequences = 23) were downloaded from GenBank and aligned to design LAMP primers for positive controls.

To perform LAMP experiments, we homogenized squid tissues (≤5 mg) with a sterilized plastic pestle, and DNA was extracted using the InstaGene Matrix (Bio-Rad, München, Germany), following the manufacturer’s instructions. With this protocol, DNA was rapidly extracted (approximately 15 min) and required no further purification steps prior to perform LAMP experiments.

### 2.2. Colorimetric LAMP Reaction

Colorimetric LAMP reactions were performed in a Bio-Rad T100 Thermal Cycler. Specifically, 4 µL of genomic DNA (≈25 ng/µL) was used as a template and mixed to 21 µL of reaction mix, containing 0.2 µM of forward outer primer (F3) and backward outer primer (B3), 1.6 µM of forward inner primer (FIP) and backward inner primer (BIP), 1.2 µM of forward loop primer (LF) and backward loop primer (LB) (Integrated DNA Technologies, Coralville, IA, USA), 0.6 M betaine (VWR International S.R.L., Milano, Italy), 1x LAMP reaction buffer (New England BioLabs, Ipswich, MA, USA), 6 mM MgSO_4_, 1.4 mM of each deoxyribonuclotide triphosphate (dNTP) (Promega, Madison, WI, USA), 0.32 U/µL Bst 2.0 WarmStart DNA polymerase (New England BioLabs, Ipswich, MA, USA), and 0.06 mM Xylenol Orange (Sigma-Aldrich-Merck, St. Louis, MO, USA). The colorimetric reaction was performed at 63 °C and the result was visualized after 30 min.

### 2.3. Fluorimetric LAMP Reaction

Fluorimetric LAMP reactions were performed in an Applied Biosystems StepOne Real-Time PCR System. Specifically, 4 µL of genomic DNA (≈25 ng/µL) were used as a template and mixed to 21 µL of reaction mix, containing 0.2 µM of F3/B3, 1.6 µM of FIP/BIP, 1.2 µM of LF/LB (Integrated DNA Technologies, Coralville, IA, USA), 0.6 M betaine (VWR International S.R.L., Milano, Italy), 1x LAMP reaction buffer (New England BioLabs, Ipswich, MA, USA), 6 mM MgSO_4_, 1.4 mM of each dNTP (Promega, Madison, WI, USA), 0.32 U/µL Bst 2.0 WarmStart DNA polymerase (New England BioLabs, Ipswich, MA, USA), and 0.2x SYBR Green (Thermo Fisher Scientific, Waltham, MA, USA). The reaction was performed at 63 °C for 60 min.

### 2.4. LAMP-AGE Procedure

Colorimetric LAMP products were electrophoresed on a 1% agarose gel to assess the amplification reactions. In detail, 500 mg of ultra-pure agarose (Thermo Fisher Scientific, Waltham, MA, USA) was dissolved in 50 mL of 1x tris-borate-EDTA buffer (TBE) (Sigma-Aldrich-Merck, St. Louis, MO, USA) using a microwave oven. A total of 5 µL of SYBR Safe DNA gel stain (Thermo Fisher Scientific, Waltham, MA, USA) was added to the solution, which then was poured into the plate with a spacer for gel polymerization. Each product of reaction was analyzed by mixing 5 µL of LAMP products to 1 µL of gel loading dye. A 0.1–10 Kb DNA ladder (New England BioLabs, Ipswich, MA, USA) was used as a molecular weight marker. The electrophoresis was performed at 80 V, and the result of the run was visualized in a Bio-Rad Gel Doc XR system.

## 3. Results and Discussion

The species substitution of labelled *Loligo vulgaris* products is representative of an important issue in the field of seafood since the global demand for cephalopods has shown a positive trend in the last decades, with the main importers and consumers being Italy, Spain, and Japan ((Food and Agriculture Organization, FAO 2016). Given this growing market importance, the correct identification and labelling of traded cephalopods is of primary importance for food safety issues due to the possible occurrence of direct or indirect toxicity phenomena in different species [34,35].

**Figure 1:**
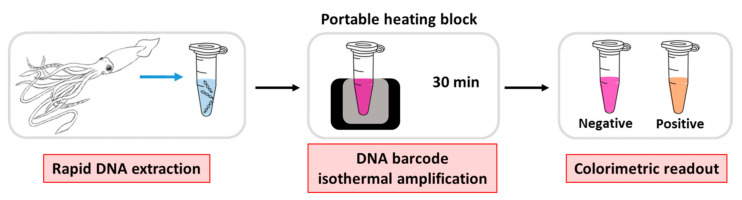
Schematic procedure of colorimetric loop-mediated isothermal amplification (LAMP) for cephalopod authentication. The proposed method consists of three steps: a rapid DNA extraction; the DNA barcode isothermal amplification process, performed at 63 °C for 30 min; and the colorimetric readout, immediately visible at the end of the reaction.

The schematic procedure of the entire method is displayed in Figure 1.

A rapid DNA extraction from the squid tissue was performed using the InstaGene Matrix (Bio-Rad, München, Germany) following the manufacturer’s instructions. Less than 5 mg of *Loligo* tissue was used as starting material prior to homogenization with a sterilized plastic pestle. Then, without any purification steps, a few microliters of the extracted target were directly added to the reaction tube, containing all reagents for the amplification reaction. The six primers were designed to be highly specific to the authentic species (unlike the polymorphic variants), and annealed to the selected regions in the presence of the target, triggering the amplification process. The colorimetric result is directly visualized by the naked-eye, due to a color change from purple to pink/orange when amplicons are produced. Remarkably, the overall colorimetric method, including the DNA extraction, the amplification reaction, and the colorimetric readout of the result, requires no instrumentation, apart from a simple heating block.

The key point of the entire strategy relies on the accurate design of species-specific LAMP primers, which target the mitochondrial COI gene of *L. vulgaris*, and allow its discrimination from the two closely related species. In detail, all the available DNA barcodes of each *Loligo* species and close relatives (Table S1) were aligned using MUSCLE online [33]. Thus, six LAMP primers (Table 1) were designed to fulfil some general requirements (such as G+C content, annealing temperature, absence of dimers, etc.) and two additional criteria, in order to improve specificity and speed up the amplification mechanism. The six specific primers were designed to target the largest number of interspecies polymorphisms. In particular, the specificity was enhanced by including at least two mismatches per primer, possibly purine/pyrimidine, to increase the melting temperature gap. In addition, where possible, one mismatch was placed at the 3′ end of the primer to hamper the DNA polymerase recognition. Such rules were revealed to be essential for the two inner primers (FIP and BIP), which are responsible for triggering the linear phase of the reaction, as well as for the two loop primers, which accelerate the amplification process [26]. Furthermore, primers were designed on the shortest and highly polymorphic region of the barcode sequence to accelerate the production of the shortest “dumbbell” amplicon, which represents the starting material for the exponential phase of the reaction.

**Figure 2:**
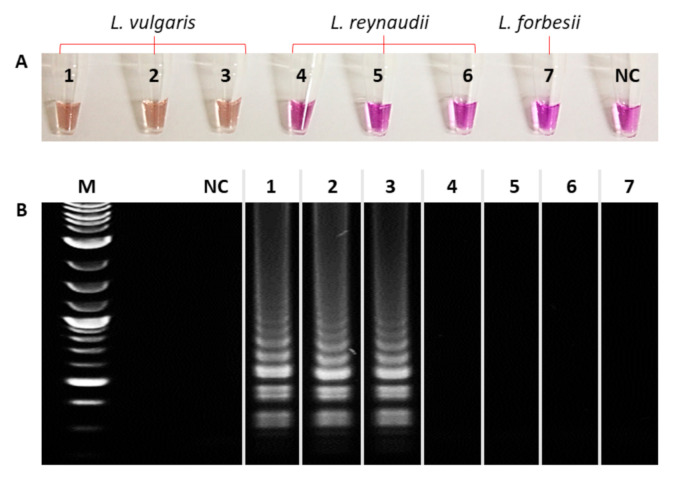
Representative colorimetric LAMP results for *L. vulgaris* authentication, applied on samples. **1**–**3**: *L. vulgaris*, **4**–**6**: *L. reynaudii*, **7**: *L. forbesii*, **NC**: pure water. (**A**) Colorimetric result. (**B**) 1% agarose gel; **M**: molecular weight marker.

The one-step colorimetric LAMP was applied to various genomic DNA samples, rapidly extracted from the tissues of *L. vulgaris*, *L. reynaudii*, and *L. forbesii* specimens, using 100 ng/µL of each crude DNA extract. The isothermal amplification process was performed in a simple heating block, and the colorimetric result was rapidly and easily visible within 30 min (Figure 2A).

As is shown, the colorimetric assay was able to correctly identify the target species, which exhibited a clear purple-to-orange color change, whilst the other species maintained the same initial color as the control sample. The reaction was further controlled by agarose gel electrophoresis, confirming that the amplification occurred specifically for *L. vulgaris*, as shown by the typical profile of LAMP products, unlike the closely related species that were not amplified (Figure 2B). In addition, a set of primers specific for a conserved region of 16S ribosomal RNA were designed as a positive control of the colorimetric LAMP (Table 2) by using the 16S rRNA sequences listed in Table S1.

**Figure 3:**
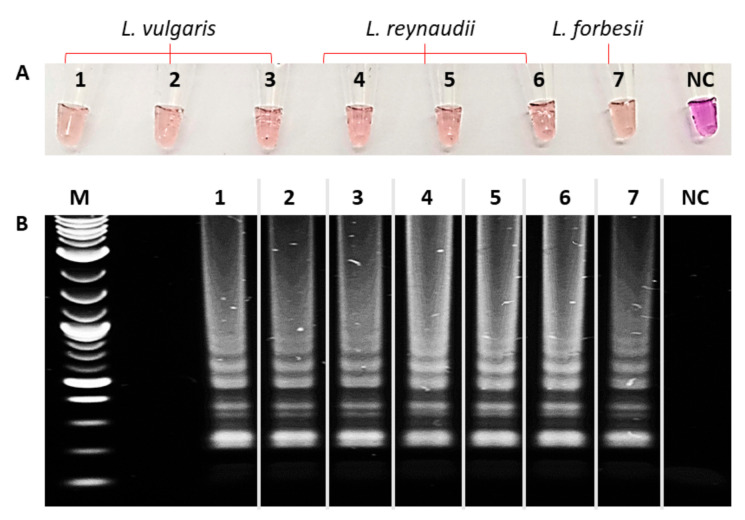
Colorimetric LAMP positive control applied on samples. **1**–**3**: *L. vulgaris*, **4**–**6**: *L. reynaudii*, **7**: *L. forbesii*, **NC**: pure water. (**A**) Colorimetric result. (**B**) 1% agarose gel; **M**: molecular weight marker.

Thus, the positive control reaction was tested on each sample under the same conditions. The visual inspection of the results showed that all samples turned pink/orange (Figure 3), demonstrating the good performance of the reaction, as well as the efficiency of the extraction protocol.

**Figure 4:**
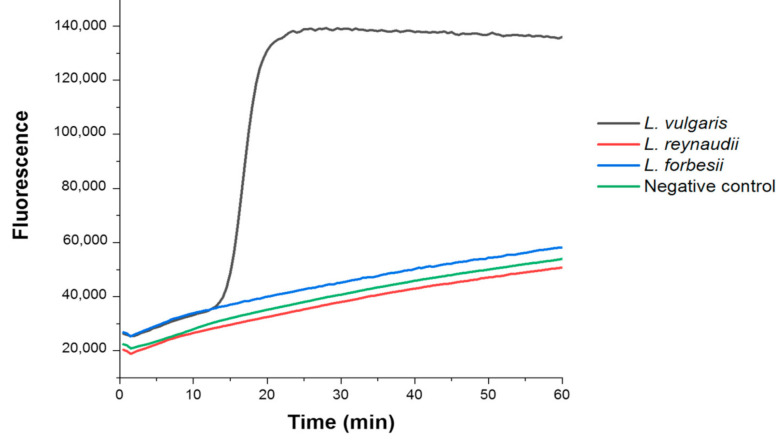
Fluorimetric LAMP for *L. vulgaris* authentication, applied on samples *L. vulgaris* (black line), *L. reynaudii* (red line), and *L. forbesii* (blue line).

Besides on-site assessments, this reaction may also represent an interesting laboratory-based methodology due to its simplicity and rapidity. Actually, when coupled to a real-time fluorescence reader, using a classical fluorescent intercalating reporter instead of a colorimetric dye, the assay exhibited high discrimination efficiency and ultra-rapidity, being capable of providing clear authentication results in only 15 min (Figure 4).

Specifically, we analyzed three genomic DNA extracts from *L. vulgaris*, *L. reynaudii*, and *L. forbesii* specimens by fluorimetric LAMP, observing fast amplification after 15 min of reaction only in the case of the target species *L. vulgaris*. Hence, the optimized design of the LAMP primers, combined with the DNA barcoding approach, enabled us to address the authentication of *L. vulgaris*. Notably, both the colorimetric and the fluorimetric LAMP were also proven effective when associated with a rapid DNA extraction procedure.

## 4. Conclusions

In this work, we reported the development of a fast, on-site assay for the authentication of squid species, addressing the specific discrimination of one species from two genetically similar and less valuable ones. By coupling the LAMP technique with DNA barcoding and rapid DNA extraction procedures for a one-step readout, we showed the possibility of achieving fast, naked-eye readout of the species discrimination. Such features make these methodologies suitable for large-scale screenings and on-site industrial quality controls in several fields.

## Figures and Tables

**Table 1:** LAMP primer sequences for *Loligo vulgaris* authentication and its discrimination from *Loligo reynaudii* and *Loligo forbesii*.

Name	Sequence
**F3**	5′-ACTGTTAAATGACGATCAACTATAC-3′
**B3**	5′-GGCTAAATCTACAGACGGT-3′
**FIP**	5′-AGCGAAGGGGGAAGTAATCAAACGGAAACTGATTAGTGCCTA-3′
**BIP**	5′-TCTGCTGTAGAAAGAGGAGCTGCTGCGTGAGAAAGATTTCTAGA-3′
**LF**	5′-CTATATCTGGCGCACCTAGTATTA-3′
**LB**	5′-GTACAGGATGAACAGTCTACCC-3′

**Table 2:** LAMP 16S rRNA primer sequences for positive control of *L. vulgaris*.

Name	Sequence
**F3-pc**	5′-TCCCTATGGTAACTATATTATAAGCA-3′
**B3-pc**	5′-ACAGCTGCGGTATTTTAAC-3′
**FIP-pc**	5′-ATTTTCATAGTGAAAAAGCTTGAATTTTTTAAAGGTCCTTAATCACCCCAATTAAAATTTATATAT-3′
**BIP-pc**	5′-TTTCTAAAAAATAAAATAGAGACAGATTAACCTTCGTGTACTAAGGTAGCATAATAATTTGCC-3′
**LF-pc**	5′-GACGAGAAGACCCTACTGAG-3′
**LB-pc**	5′-CAAACCATTCATTCTAGCCTCAAATTAT-3′

## Supplementary Materials

The following are available online at https://www.mdpi.com/2079-6374/10/12/190/s1: Table S1: *Loligo* spp. and close relatives cytochrome c oxidase subunit I (COI) and 16S ribosomal RNA sequences used to design LAMP primers.
