# Supplementary material for: A Rapid Colorimetric Assay for On-Site Authentication of Cephalopod Species
Source: Biosensors (Basel). 2020 Nov 24;10(12):190. doi: 10.3390/bios10120190 (PMC7760856; doi:10.3390/bios10120190)
Supplement: Supplementary file 1 [file biosensors-10-00190-s001.pdf]

biosensors

MDPI

Supporting Information

A rapid colorimetric assay for on-site authentication of cephalopod species

Giuseppina Tatulli [1,§], Paola Cecere [1,§], Davide Maggioni [2,3], Andrea Galimberti [4,*], and Pier Paolo Pompa [1,*]

[1] Istituto Italiano di Tecnologia, Nanobiointeractions&Nanodiagnostics, Via Morego 30 – 16163 Genova, Italy; giuseppina.tatulli@iit.it (G.T.); paola.cecere@iit.it (P.C.)

[2] Department of Earth and Environmental Sciences (DISAT), University of Milano - Bicocca, P.za Della Scienza 1, 20126-I Milan, Italy; davide.maggioni@unimib.it (D.M.)

[3] Marine Research and High Education (MaRHE) Center, University of Milano - Bicocca, 12030 Faafu Magoodhoo, Maldives

[4] ZooPlantLab, Department of Biotechnology and Biosciences, University of Milano - Bicocca, P.za Della Scienza 2, 20126-I Milan, Italy

§ These authors contributed equally to this work

* Correspondence: andrea.galimberti@unimib.it (A.G.); pierpaolo.pompa@iit.it (P.P.P.)

Table S1. *Loligo* spp. and close relatives COI and 16S rRNA sequences used to design LAMP primers.

**Species**	**GenBank accession numbers (COI)**	**GenBank accession numbers (16S rRNA)**
Loligo vulgaris	AF075397, JQ623949, KC311393-KC311395, KC789174-KC789193, KF369141-KF369144, KF854075, KF854076, KM517926-KM517928, MH292979, MH293012, MH293014, MH293016, MH293017, MH293037, MH293038, MH293041-MH293044, MH293066, MH293071-MH293074, MH293083, MH293084, MH293087, MH293091, MH293099, MH293106, MH293108, MH473337, MH473339	KF854037, KF854038, LC310709, X79585
Loligo forbesii	AF075402, KF854077, KF854078, KM517903-KM517925, MH293018, MH293090	AF110075, KF854039, KF854040, X79583
Loligo reynaudii	AF075406, MH293021, MH293022, MH293029, MH293045, MH293046, MH293052, MH293053, MH293056-MH293065,	KF854035, KF854036
Alloteuthis subulata	AF075387, EU668098-EU668100, GU327599	AF110072
Doryteuthis gahi	AF075399, KF854066-KF854068	AF110076, AJ000106
Doryteuthis opalescens	AF000051, AF075395, JF730438-JF730440	AF110077, AJ000107
Doryteuthis pealeii	AF207910-AF207914	AF110079, AY686590
Doryteuthis pleii	AF207934-AF207938	AF110080
Heterololigo bleekeri	AB441180-AB441183, AF075388	AF110074
Uroteuthis chinensis	EU349429-EU349433	AF369955, AJ000105, EU234588, LC121051

© 2020 by the authors. Submitted for possible open access publication under the terms and conditions of the Creative Commons Attribution (CC BY) license (http://creativecommons.org/licenses/by/4.0/).
